# Rationale and design of the Children's Oncology Group study AAML1831 integrated cardiac substudies in pediatric acute myeloid leukemia therapy

**DOI:** 10.3389/fcvm.2023.1286241

**Published:** 2023-12-01

**Authors:** Kasey J. Leger, Nora Robison, Hari K. Narayan, Amanda M. Smith, Tenaadam Tsega, Jade Chung, Amber Daniels, Zhen Chen, Virginia Englefield, Biniyam G. Demissei, Benedicte Lefebvre, Gemma Morrow, Ilona Dizon, Robert B. Gerbing, Reena Pabari, Kelly D. Getz, Richard Aplenc, Jessica A. Pollard, Eric J. Chow, W. H. Wilson Tang, William L. Border, Ritu Sachdeva, Todd A. Alonzo, E. Anders Kolb, Todd M. Cooper, Bonnie Ky

**Affiliations:** ^1^ Division of Pediatric Hematology/Oncology, Seattle Children’s Hospital, University of Washington, Seattle, WA, United States; ^2^ Division of Cardiology, Department of Pediatrics, Rady Children’s Hospital San Diego, University of California San Diego, La Jolla, CA, United States; ^3^ Division of Cardiology, Perelman School of Medicine, University of Pennsylvania, Philadelphia, PA, United States; ^4^ Division of Cardiology, Children’s Healthcare of Atlanta, Atlanta, GA, United States; ^5^ Division of Cardiology, Seattle Children’s Hospital, Seattle, WA, United States; ^6^ Children’s Oncology Group, Monrovia, CA, United States; ^7^ Division of Hematology/Oncology, The Hospital for Sick Children, Toronto, ON, Canada; ^8^ Division of Oncology, Children’s Hospital of Philadelphia, University of Pennsylvania Perelman School of Medicine, Philadelphia, Pennsylvania; ^9^ Division of Pediatric Oncology, Dana-Farber Cancer Institute, Boston, MA, United States; ^10^ Department of Pediatrics, Harvard Medical School, Boston, MA, United States; ^11^ Clinical Research and Public Health Sciences Divisions, Fred Hutchinson Cancer Center, Seattle, WA, United States; ^12^ Department of Cardiovascular Medicine, Heart Vascular and Thoracic Institute, Cleveland Clinic, Cleveland, OH, United States; ^13^ Department of Pediatrics, Emory University School of Medicine, Atlanta, GA, United States; ^14^ Keck School of Medicine, University of Southern California, Los Angeles, CA, United States; ^15^ Nemours Center for Cancer and Blood Disorders, Alfred I. DuPont Hospital for Children, Wilmington, DE, United States

**Keywords:** pediatric acute myeloid leukemia (AML), liposomal anthracycline, CPX-351, dexrazoxane, cardiotoxicity, risk prediction, cardiac biomarkers

## Abstract

**Background:**

Pediatric acute myeloid leukemia (AML) therapy is associated with substantial short- and long-term treatment-related cardiotoxicity mainly due to high-dose anthracycline exposure. Early left ventricular systolic dysfunction (LVSD) compromises anthracycline delivery and is associated with inferior event-free and overall survival in *de novo* pediatric AML. Thus, effective cardioprotective strategies and cardiotoxicity risk predictors are critical to optimize cancer therapy delivery and enable early interventions to prevent progressive LVSD. While dexrazoxane-based cardioprotection reduces short-term cardiotoxicity without compromising cancer survival, liposomal anthracycline formulations have the potential to mitigate cardiotoxicity while improving antitumor efficacy. This overview summarizes the rationale and methodology of cardiac substudies within AAML1831, a randomized Children's Oncology Group Phase 3 study of CPX-351, a liposomal formulation of daunorubicin and cytarabine, in comparison with standard daunorubicin/cytarabine with dexrazoxane in the treatment of *de novo* pediatric AML.

**Methods/design:**

Children (age <22 years) with newly diagnosed AML were enrolled and randomized to CPX-351-containing induction 1 and 2 (Arm A) or standard daunorubicin and dexrazoxane-containing induction (Arm B). Embedded cardiac correlative studies aim to compare the efficacy of this liposomal anthracycline formulation to dexrazoxane for primary prevention of cardiotoxicity by detailed core lab analysis of standardized echocardiograms and serial cardiac biomarkers throughout AML therapy and in follow-up. In addition, AAML1831 will assess the ability of early changes in sensitive echo indices (e.g., global longitudinal strain) and cardiac biomarkers (e.g., troponin and natriuretic peptides) to predict subsequent LVSD. Finally, AAML1831 establishes expert consensus-based strategies in cardiac monitoring and anthracycline dose modification to balance the potentially competing priorities of cardiotoxicity reduction with optimal leukemia therapy.

**Discussion:**

This study will inform diagnostic, prognostic, preventative, and treatment strategies regarding cardiotoxicity during pediatric AML therapy. Together, these measures have the potential to improve leukemia-free and overall survival and long-term cardiovascular health in children with AML.

**Clinical trial registration:** https://clinicaltrials.gov/, identifier NCT04293562

## 1. Background

Anthracycline chemotherapy is an effective component of pediatric acute myeloid leukemia (AML) therapy. However, cardiotoxicity secondary to anthracyclines compromises short- and long-term cardiovascular health along with therapy delivery and leukemia-free survival. Late anthracycline cardiomyopathy, a commonly recognized phenomenon, is seen in more than 15% of childhood AML survivors and is associated with high rates of cardiac transplantation and mortality (1–3). More recent data demonstrates the high prevalence and significant implications of early cardiotoxicity manifested as left ventricular systolic dysfunction (LVSD) during or soon after AML therapy (4, 5). Among 1,022 children with *de novo* AML treated in the COG phase 3 randomized trial, AAML0531, 12% experienced LVSD during and within 18 months of completing protocol therapy [defined as a left ventricular ejection fraction (LVEF) <50% or fractional shortening (LVFS) <24%] (5). Although some cases of cardiac dysfunction were transient, patients who developed LVSD during protocol therapy were much more likely to experience LVSD during the 5-year follow-up period (HR, 12.1, 95% CI, 4.2–34.8). Additional data on childhood leukemia survivors also support the association between early cardiotoxicity during therapy and late cardiomyopathy (6, 7). Importantly, early LVSD is also associated with decreased leukemia-free and overall survival (OS). Children on AAML0531 who developed LVSD during or soon after therapy had a 5-year event-free survival (EFS) of 32%, significantly lower than the 48% EFS observed in those without LVSD (*p* = 0.017). OS was also reduced in patients with LVSD (50% vs. 67%, *p* = 0.004) (5). The AAML0531 protocol included instructions to omit anthracycline following incident LVSD; thus, these survival decrements are likely partly due to compromised anthracycline delivery. Notably, the 6.3% improvement in EFS observed in the gemtuzumab ozogamicin-containing arm of the randomized trial was dramatically less than the observed cardiotoxicity-related decreases in EFS and OS (8). The substantial impact of cardiotoxicity on cancer treatment delivery and survival in childhood AML underscores the importance of understanding cardiotoxicity risk factors and developing effective prevention strategies.

Thus, one of the major objectives of the ongoing Children's Oncology Group (COG) phase 3 clinical trial in *de novo* AML, AAML1831, is to prospectively evaluate cardioprotection strategies and predictors of cardiotoxicity. AAML1831 randomizes participants between standard anthracycline-containing induction therapy with dexrazoxane vs. CPX-351, a dual-drug liposomal encapsulation of daunorubicin and cytarabine in a synergistic 1:5 molar ratio (NCT04293562). The primary objective of this trial is to compare EFS across treatment arms. This overview explains the rationale and methodology of the secondary objectives: measuring and mitigating cardiotoxicity.

### 1.1. Rationale for cardioprotective strategies

Dexrazoxane is the only US Food and Drug Administration (FDA)-approved cardioprotective agent against anthracycline cardiomyopathy. The efficacy of dexrazoxane in reducing cardiotoxicity has been demonstrated in a variety of pediatric oncology settings, including pediatric AML (4, 9–11). Within the COG phase 3 trial, AAML1031, dexrazoxane was administered at the discretion of the treating physician. Compared with dexrazoxane unexposed (*n* = 918), dexrazoxane exposed (*n* = 96) had smaller declines in ejection fraction (EF) (*p* < 0.05), an overall lower risk for early LVSD (6% vs. 19%, *p* = 0.005), and a tendency toward a greater likelihood of LVSD resolution (4). Importantly, each of these pediatric studies (many with late follow-up) showed no differences in the rates of relapse, secondary malignancy, or mortality associated with dexrazoxane (12–14).

Liposomal anthracycline delivery systems are another promising strategy to mitigate cardiotoxicity while maintaining anti-tumor efficacy (15–18). Liposomal encapsulation of anthracycline reduces its ability to penetrate the tight capillary junctions of the heart allowing for preferential accumulation in the tumor (19). Meta-analyses of adult cancer trials of liposomal anthracyclines show >50% reduction in heart failure compared to standard anthracyclines (20, 21). The International Berlin-Frankfurt-Münster Study Group demonstrated the safety of intensifying anthracycline through the use of liposomal daunorubicin without increasing toxicity in children with newly diagnosed AML (22). CPX-351 is a liposomal formulation of daunorubicin+cytarabine (DA) contained within a liposome at a fixed 5:1 molar ratio, allowing for ratio-dependent synergy of the drug combination and prolonged plasma drug levels (23–26). CPX-351 was FDA-approved in August 2017 for adults with therapy-related AML or AML with myelodysplasia-related changes based on a phase 3 trial demonstrating superior OS in patients treated with CPX-351 compared to those treated with DA (HR = 0.69; 95% CI, 0.52–0.90; one-sided *p* = 0.003) (27). In 2021, the CPX-351 label was expanded to include pediatric patients based on safety and efficacy data from two early-phase pediatric trials, namely, AAML1421 and CPX-MA-1201 (28, 29).

Although the cardioprotective effects of CPX-351 are not fully understood, adult studies suggest a potential reduction in cardiotoxicity compared with standard daunorubicin and cytarabine. A randomized study of CPX-351 vs. daunorubicin/cytarabine in over 300 older adults with AML demonstrated superior efficacy and lower rates of cardiotoxicity with CPX-351 (27, 30). A post hoc analysis of centrally quantitated echocardiograms from 102 adults treated in this trial demonstrated lower rates of clinically significant LVEF decline (>10% decrease from baseline and LVEF < 53%) or GLS decline (>12% relative decrease from baseline and GLS > −18%) in patients treated with CPX-351 vs. daunorubicin/cytarabine (9% vs. 20% and 21% vs. 44%, respectively) at a median follow-up of approximately 6 months (30). In a phase I/II COG study of CPX-351 in the first relapse of pediatric AML, grade 2+ LVSD occurred in 7 of 38 (18%) heavily anthracycline pretreated children who received CPX-351 followed by fludarabine/cytarabine (28). There is a lack of data on how this is compared to standard anthracycline or non-anthracycline salvage regimens. A randomized study in anthracycline-naive patients is the optimal setting for a prospective evaluation of the cardioprotection of liposomal anthracyclines. Hence, the rationale for an ongoing randomized phase 3 study of CPX-351 in children with *de novo* AML is described herein. If liposomal anthracyclines can mitigate cardiotoxicity while maintaining or even enhancing leukemia-free survival, they could substantially improve the therapeutic options for AML.

In addition, given the high degree of cardiotoxicity associated with mitoxantrone relative to other anthracycline derivatives (31), AAML1831 aims to limit mitoxantrone exposure in upfront AML therapy. Given that AAML1031 did not show benefit to the intensification of high-risk AML therapy with mitoxantrone and cytarabine during induction 2, high-risk subjects will no longer receive mitoxantrone prior to hematopoietic stem cell transplant (HSCT). Because children with low-risk cytomolecular features who are MRD negative after induction 1 are a favorable subgroup where the risk of late cardiotoxicity may outweigh the benefit of mitoxantrone, AAML1831 replaces mitoxantrone/cytarabine in Intensification II with Capizzi II cytarabine for this subset of patients to maintain the intensity of therapy with less cardiotoxicity risk.

### 1.2. Rationale for selected cardiac variables and end points

#### 1.2.1. Primary cardiac endpoint

Routine cardiac monitoring during pediatric AML therapy typically consists of serial precycle echocardiography to assess left ventricular (LV) dysfunction, manifested as declines in LVEF and fractional shortening (LVFS). Although the linear measure of LVFS has a long historical precedent in pediatrics due to its ease of measurement, it is not recommended for cardiotoxicity surveillance due to its strong dependence on LV geometric assumptions and poor reliability between acquisitions (32, 33). In contrast, 2D echocardiography-derived LVEF captures the volumetric change in LV chamber dilation between diastole and is a more reliable measure of LV function. Existing pediatric data demonstrate a striking incidence of LVEF declines during AML therapy and their association with inferior leukemia-free survival and worse cardiac outcomes on late follow-up (6, 7). Furthermore, LVEF decline is a robust predictor of cardiac outcomes in the general population (34). Thus, the primary measure of cardiac outcome for assessment of cardiotoxicity across arms is based on serial LVEF measurements during therapy and 1 year after the end of therapy.

#### 1.2.2. Secondary cardiac measures

The application of newer metrics, such as blood biomarkers and novel echo indices, is an area of active investigation to identify vulnerable patients prior to overt and/or irreversible cardiac remodeling and dysfunction (35, 36). Therefore, AAML1831 will utilize sensitive echocardiographic measures and cardiac biomarkers to (a) compare the impact of cardiotoxic therapies across arms and (b) complement patient- and treatment-specific variables to identify individuals at high cardiovascular risk.

i. *Strain echocardiography* is a sensitive measure of cardiac deformation that offers direct insight into myocardial mechanics and contractility (37). Studies performed in a variety of heart failure populations suggest that global longitudinal strain (GLS) is the first dimension of myocardial deformation to become impaired, potentially reflecting the vulnerability of the subendocardium (38). Numerous adult oncology studies have shown that early GLS declines predict subsequent LVEF declines (39–41). GLS declines have also been observed in childhood cancer in both the acute and long-term follow-up setting after anthracycline therapy (26, 42–44). Collectively, these findings indicate a likely role for strain as a meaningful early measure of cardiotoxicity during childhood AML therapy and support its inclusion as a sensitive assessment of cardiac function (outcome variable, cardiac aim 2) and an early predictor of more advanced declines in LVEF (predictor variable, cardiac aim 3).
ii. *Cardiac biomarkers:* Pediatric studies have demonstrated the prevalence of troponin and natriuretic peptide elevation during anthracycline therapy and their association with subsequent adverse cardiac remodeling (45). In addition, early troponin elevations in adult cancer patients predict subsequent cardiac events (46). Although these studies suggest the utility of troponin for early detection of anthracycline-induced cardiac injury, the specific patterns of biomarker elevations that predict progression to heart failure have not yet been determined in children. Therefore, serial biomarker analysis may help clarify the role of these blood biomarkers in predicting cardiotoxicity. Although there may be differences in troponin T as compared to troponin I in the cancer population, troponin T has been extensively studied in the pediatric population, and both markers are associated with prognosis in the cardiovascular population (45, 47–49). Moreover, blood will also be banked for future assays.

### 1.3. Rationale for LVSD case definition

Definitions of cardiotoxicity vary according to clinical trials and guidelines. The most commonly accepted definition of clinically significant cardiotoxicity in adults is a >10% decrease in LVEF to an LVEF < 50% (50). Baseline cardiac dysfunction or cardiovascular comorbidities are uncommon in pediatrics, thus pediatric definitions commonly rely on an absolute LVEF or LVFS without taking into account the decline from baseline. The Common Terminology Criteria for Adverse Events (CTCAE) definitions of LVSD have varied over time but have generally defined grade 2 or higher LV dysfunction using an LVEF threshold of 50% (termed “ejection fraction decreased” in CTCAE v. 5). Therefore, LVSD cases will be defined by an LVEF < 50% for all AAML1831 cardiac analyses. The first cardiac aim (described below) includes all subjects treated on Arms A/B using site-measured/reported LVEF to determine LVSD (*n* = ∼1,068). The second cardiac aim is limited to a subcohort of representative subjects who will undergo detailed cardiac phenotyping via core lab echocardiography quantitation and serial cardiac biomarker assessment (*n* = ∼510).

## 2. Cardiac aims

The AAML1831 cardiac studies were designed to address the high rate of cardiotoxicity in children who have undergone treatment for AML. The methods and planned analyses were specifically designed to address several secondary and exploratory study aims included in AAML1831.

First, to compare the cardioprotective efficacy of dexrazoxane vs. liposomal anthracycline encapsulation, the AAML1831 cardiac studies will compare the incidence of LVSD in children with non-FLT3 mutant *de novo* AML treated with standard daunorubicin and dexrazoxane-containing induction (Arm A) vs. CPX-351-containing induction (Arm B) (cardiac aim 1). Changes in serial centrally quantitated, echocardiography-derived measures of cardiac function (including LVEF and GLS) and biomarkers of cardiomyocyte stress and injury (including natriuretic peptides and troponin) will be similarly compared across arms A vs. B (cardiac aim 2). To identify predictors of cardiotoxicity, AAML1831 analyses will determine if early changes in sensitive echocardiographic measures of cardiac function (e.g., GLS and elevation of circulating cardiac biomarkers) are associated with subsequent declines in LVEF (cardiac aim 3). An exploratory aim of cardiac studies is to quantify associations between host factors (age, sex, BMI, race), treatment exposures (cumulative anthracycline dose, anthracycline arm, HSCT vs. chemotherapy alone), early declines in GLS, and elevations in cardiac biomarkers (high sensitivity troponin T (hs-cTnT) and N-terminal pro-B-type natriuretic peptide (NT-proBNP)) with subsequent LVSD. These data will then be used to develop a multi-marker risk prediction model incorporating significant host factors, treatment exposures, and echocardiographic predictors for the development of LVSD within 1 year of completing therapy. These aims will be restricted to the population of non-FLT3 mutant subjects treated on Arms A/B, whereas those treated with concurrent tyrosine kinase inhibition on Arms C/D will be analyzed separately.

## 3. Methods

### 3.1. Subjects

Eligible patients included those aged less than 22 years at the time of study enrollment with newly diagnosed AML according to the 2016 WHO classification (51). The subjects must also have one of the following: >20% bone marrow blasts, <20% bone marrow blasts with one or more defining genetic abnormalities listed by the study, or at least 1,000/µl circulating leukemic cells. The subjects with cardiac dysfunction, defined as LVEF <50% or LV shortening fraction less than 24%, are not eligible to participate. Additional exclusion criteria include germline AML predisposition syndrome, concurrent malignancy, or any of the following myeloid leukemias: juvenile myelomonocytic leukemia, Philadelphia chromosome-positive AML, mixed-phenotype acute leukemia, acute promyelocytic leukemia, AML arising from myelodysplasia, or therapy-related myeloid neoplasms. Receipt of prior anti-cancer therapy is prohibited unless specified in the protocol. All patients and/or their parents or legal guardians must provide written informed consent.

### 3.2. Intervention/trial design

In this phase 3 COG study, children and adolescents with newly diagnosed AML were randomized 1:1 at enrollment to receive standard daunorubicin and cytarabine-containing induction therapy with dexrazoxane vs. CPX-351. All patients will undergo two induction cycles with either daunorubicin (50 mg/m^2^ on days 1, 3, and 5 of each cycle)/cytarabine or CPX-351 (135 units/m^2^/dose, equal to 60 mg/m^2^/dose of daunorubicin on days 1, 3, and 5 of induction and 115 units/m^2^/dose, equal to 50 mg/m^2^ of daunorubicin on days 1, 3, and 5 of induction 2).

**Figure 1:**
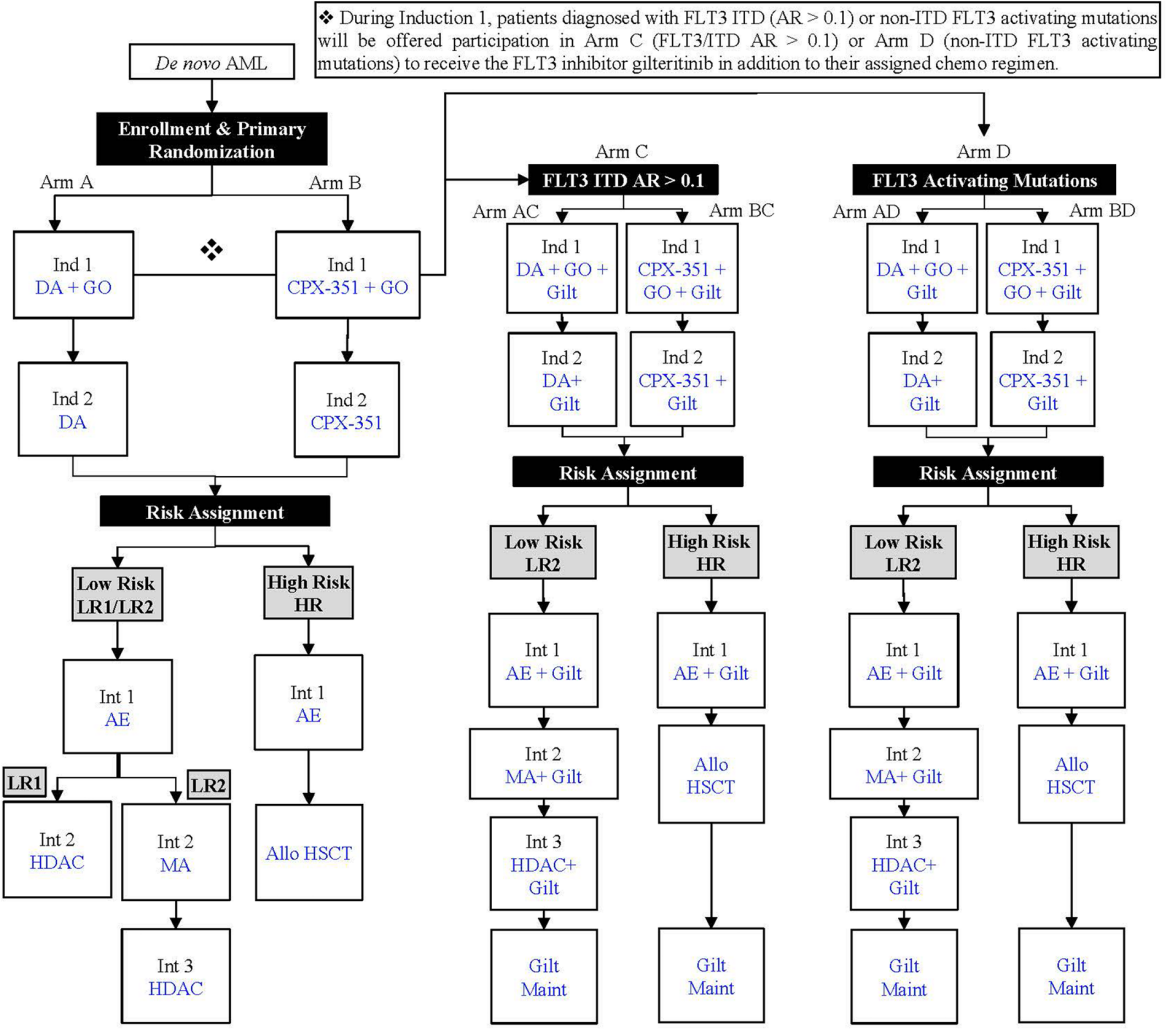
AAML1831 study schematic. AE, cytarabine/etoposide; Allo, allogeneic; AR, allelic ratio; DA, daunorubicin/cytarabine; DL, dose level, Gilt, gilteritinib; GO, gemtuzumab ozogamicin; HDAC, high-dose cytarabine/asparaginase (Capizzi II); HSCT, hematopoietic stem cell transplant; Ind, induction; Int, intensification; ITD, internal tandem duplication; MA, mitoxantrone/cytarabine; Maint, maintenance.

Patients deemed high risk based on disease response or molecular/cytogenetic features will undergo a single intensification cycle followed by HSCT (52). Those with low-risk features will undergo 2–3 intensification cycles depending on their cytomolecular features and response. Subjects with favorable cytomolecular features and minimal residual disease <0.05% at the end of induction 1 are assigned to the lowest-risk arm [low risk 1 (LR1)]. LR1 subjects will receive only two intensification cycles without mitoxantrone. Patients whose AML harbors FLT3 internal tandem duplication or activating mutations will also be offered participation in arms that add the tyrosine kinase inhibitor gilteritinib (Arms C/D). An overview of therapeutic agents included in each induction and intensification cycle is shown in the study schematic (Figure 1). All patients will enter follow-up and continue to submit cardiac AEs, LVEF/LVFS data, and DICOM echocardiographic images for 10 years after completion of protocol therapy unless they are removed from the study due to death, loss of contact, withdrawal of consent, or enrollment on another COG anti-cancer therapeutic trial. The current clinical trial was approved by the Pediatric Central Institutional Review Board (CIRB) and the COG Scientific Council.

### 3.3. Evaluations

#### 3.3.1. Echocardiograms

**Figure 2:**
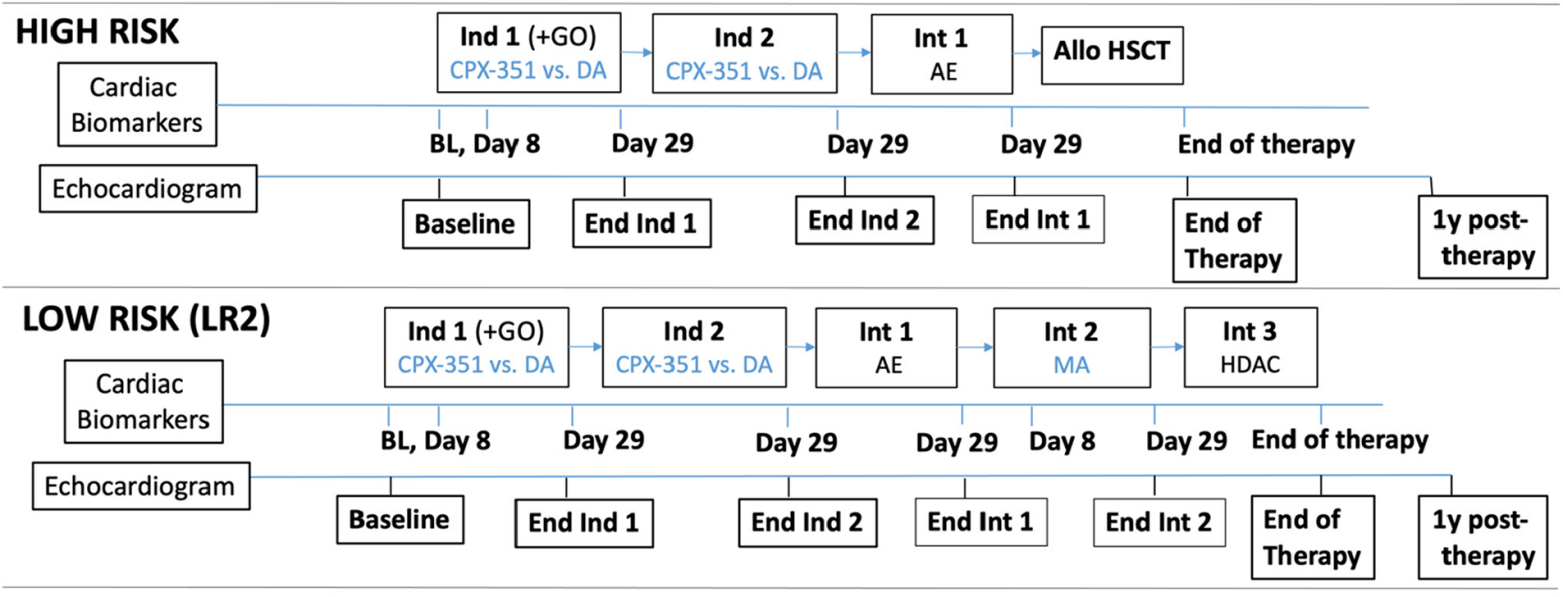
AAML1831 cardiac studies schematic. Cardiac biomarker and echocardiogram time points for subjects treated on Arms A/B (without concurrent gilteritinib). Anthracycline agents are denoted in blue. AE, cytarabine/etoposide; Allo, allogeneic; BL, baseline; DA, daunorubicin/cytarabine; GO, gemtuzumab ozogamicin; HDAC, high-dose cytarabine/asparaginase (Capizzi II); HSCT, hematopoietic stem cell transplant; Ind, induction; Int, intensification; MA, mitoxantrone/cytarabine.

Cardiac function will be serially assessed by echocardiograms at baseline, following each subsequent therapy cycle, 12 months following completion of protocol therapy, and at least every 2 years thereafter through 10 years following completion of protocol therapy (Figure 2). Additional echocardiograms are required for patients with FLT3-mutant AML receiving gilteritinib maintenance, including pre-, mid- (week 25), and postmaintenance. Pre-/postcycle echocardiograms are to be performed when the patient has recovered from the toxicities of the previous chemotherapy cycle and ideally when off IV fluids and with evidence of blood count recovery. Echocardiograms are to be performed according to the AAML1831 echocardiogram acquisition protocol, reflective of a contemporary and comprehensive standard-of-care pediatric echocardiogram. Emphasized views include the 2D LV images in the apical four-chamber, two-chamber, and three-chamber views and the parasternal short-axis views at the LV apex, mid-, and basal levels. Site sonographers are recommended to review the AAML1831 echocardiogram acquisition training slides or webinar and undergo a certification process involving detailed feedback from the core lab regarding echocardiogram quality. Echocardiograms are analyzed for LV function at each site per standard-of-care to inform clinical care and safety of ongoing anthracycline delivery. Each site-measured LVEF and LVSF are required to be submitted in the AAML1831 case report forms. DICOM echocardiogram images are submitted to the Penn Center for Quantitative Echocardiography for post hoc quantitation in a blinded fashion. The primary measurements include LVEF derived by biplane Simpson's method, global longitudinal and circumferential strain, and LV wall thickness (LVWT) to LV end-diastolic dimension (LVEDD) ratio. Additional comprehensive measures of cardiac structure and function relating to chamber size, Doppler indices, and valvular disease are also obtained, which will enable important exploratory analyses of the impact of AML therapies on cardiac remodeling, systolic and diastolic function, as well as more novel indices such as regional strain (53, 54). All measures are quantitated using the Tomtec cardiac performance analysis (Unterschleissheim, Germany).

#### 3.3.2. Electrocardiograms

Electrocardiograms (ECGs) must be carried out prior to each cycle and at the end of therapy. Additional time points are required for subjects receiving gilteritinib given the risk for QT prolongation. The corrected QT value based on the Fridericia formula (QTcF) is calculated by the site and used to screen for high-grade QTc prolongation that would necessitate gilteritinib dose modification. Uncorrected QT and ventricular rate are to be submitted in the study case report forms at the required time points to facilitate central QTc tracking/review.

#### 3.3.3. Cardiac biomarkers

Plasma submission for cardiac biomarker analysis is required prior to each chemotherapy cycle, on day 8 of induction 1 and intensification 2 (in LR2 strata receiving mitoxantrone), and at the end of protocol therapy. Subjects treated on Arms C/D with gilteritinib will also undergo biomarker sample collection prior to the start of maintenance, mid-maintenance (week 25), and end of maintenance (Figure 2). Peripheral blood is to be collected in one EDTA tube and one lithium heparin tube. Biomarker assays will be performed *post hoc*; thus, results are not available to sites in real time. High-sensitivity troponin T will be analyzed at a CAP/CLIA-approved biomarker core laboratory the Cleveland Clinic's Center for Cardiovascular Diagnostics and Prevention for analysis using the Elecsys Troponin T Gen 5 STAT and NT-proBNP assays from the Cobas platform (Roche Diagnostics, Indianapolis IN). Assays are highly reproducible, standardized, and FDA-approved for clinical use with coefficients of variation of <10%. Furthermore, blood will also be banked for future assays.

#### 3.3.4. Cardiac data reporting

Cardiac adverse events (e.g., ejection fraction decrease, LVSD, heart failure, QTc prolongation) must be reported by institutions at any grade of severity via the clinical trial data portal RAVE EDC. The site measured the LVEF and LVSF of each echocardiogram, and the uncorrected QT and ventricular rate from each ECG must be reported. Adverse events are classified by site principal investigators according to the National Cancer Institute Common Terminology Criteria for Adverse Events (version 5.0).

### 3.4. Anthracycline dose modification strategy

**Figure 3:**
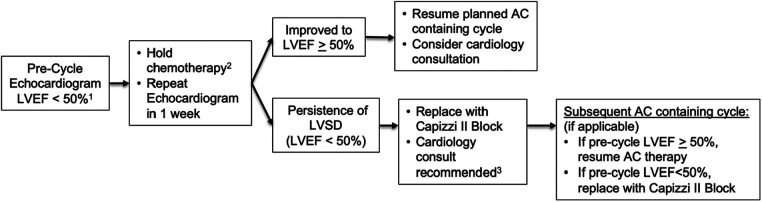
Approach to anthracycline/anthracenedione (AC) dose modification for left ventricular systolic dysfunction (LVSD) on AAML1831. ^1^EF should be measured by the biplane Simpson's method. If unevaluable, use alternate EF measurement. If EF is unevaluable, use the shortening fraction (SF) threshold of SF < 24%. ^2^Only relevant to AC-containing cycles. For low EF prior to non-AC cycles, may proceed with therapy. Recommend cardiology consultation for consideration of cardiac remodeling medications to support cardiac function. ^3^For patients with persistent LVSD, cardiology consultation is strongly recommended for consideration of cardiac remodeling medications to support the recovery of cardiac function.

AAML1831 utilizes an anthracycline dose modification strategy that aims to optimize anthracycline delivery while minimizing cardiotoxicity (Figure 3). Thus, the LVEF threshold for anthracycline dose modification is restricted to clinically significant declines in cardiac function to an LVEF below 50%. In the event of LVSD, the protocol recommends repeating the echocardiogram in 1 week to confirm cardiac dysfunction prior to proceeding with dose modification of anthracycline, if clinically appropriate. Subsequent reinitiation of anthracycline is permitted regardless of whether the episode occurred in the presence or absence of infection as long as the LVEF recovers to ≥50%. In those who require withholding of LVSD, AAML1831 maintains the intensity of protocol therapy by replacing the anthracycline-containing block with a similarly intensive high-dose cytarabine (Capizzi II) block. AAML1831 strongly recommends cardiology consultation and consideration of neurohormonal therapy, such as ACE inhibition or beta-blockade, in those with LVSD, which may help support the recovery of cardiac function (Figure 3). The receipt of cardiac remodeling medications will be collected in study case report forms and allow analysis of their impact on cardiac functional recovery.

### 3.5. Statistical analysis

#### 3.5.1. Comparison of cardiotoxicity across arms

To compare the incidence of LVSD across the entire cohort of non-FLT3 mutant AML subjects treated on Arm A (standard anthracycline plus dexrazoxane) or Arm B (CPX-351), LVSD cases will be identified based on site quantitated LVEF measured on any on-therapy or end of therapy echocardiogram. LVSD will be defined as a biplane Simpson's LVEF < 50%. If the biplane Simpson LVEF is unavailable, LVSD will be defined by a single-plane LVEF measurement, or, if also unavailable, an LVFS below 24%. A time-to-event analysis will be used to compare the cumulative incidence of LVSD between arms. LVEF and GLS declines throughout therapy will be compared across Arm A and Arm B using generalized estimating equations (GEE) including centrally quantitated LVEF and GLS performed at all precycle timepoints, end of therapy, and 12 months off therapy. Similarly, GEE incorporating baseline and post-anthracycline biomarker measurements will also be performed to compare changes in hs-cTnT and NT-proBNP throughout therapy.

#### 3.5.2. Cardiotoxicity risk prediction

For analysis of cardiotoxicity predictors, a case cohort will be constructed composed of a subcohort of sequentially enrolled patients treated on arms A and B (*n* = 510 including 84 high-risk and 171 low-risk patients per arm), plus any additional LVSD cases identified from the Arm A/B study population outside of the subcohort based on site measured LVEF and confirmed on core lab quantitation. LVSD cases will be defined as a centrally measured core lab LVEF < 50% or, if LVEF is not available, LVFS < 24% at any precycle, end-of-therapy, or off-therapy echocardiogram between intensification and 12 months posttherapy.

Among the case cohort (*n* = 543), a series of weighted ordinary least-squares linear regression models will be used to define the association between postinduction 1 GLS and the postinduction 1 change from baseline in GLS (two separate models) and LVEF at 12 months posttherapy (dependent variable). A similar series of regression models will be used to assess the association between on-therapy hs-cTnT and NT-proBNP elevations (predictors) with EF 12 months after therapy (outcome variable).

Subsequently, time-to-event survival models will be used to estimate the relationships of relevant host factors (age, sex, BMI, race) and treatment exposures (cumulative anthracycline dose, anthracycline arm, HSCT vs. chemotherapy alone) with LVSD, defined as core lab quantitated LVEF < 50% occurring on or within 1 year after protocol therapy. After the estimation of the relationship of these host and treatment variables with LVSD, a multi-marker risk prediction model will be developed incorporating significant host factors, treatment exposures, and echocardiographic predictors for the development of LVSD within 1 year of completing therapy. Finally, a separate cohort of children treated on AAML1031 will be retrospectively analyzed to provide external validation for this risk prediction model.

### 3.6. Sample size considerations

AAML1831 will enroll approximately 1,400 subjects over an estimated 5-year accrual period. The integrated cardiac studies are designed to address cardiac-specific aims within a representative subcohort of the study population with the primary emphasis on comparing the cardiotoxicity across arms. The case cohort is constructed to identify predictors of cardiotoxicity will incorporate cases from the entire study population (analyzing those treated on Arms C/D with gilteritinib separately). Estimating an LVSD rate of 8% in the conventional chemotherapy arm (inclusive of dexrazoxane) based on AAML1031 data and 5% in the CPX-351 arm, 33 cases are anticipated within the subcohort plus an additional 33 cases from the non-FLT3 mutant population (i.e., Arm A/B) outside of the subcohort (*n* = 510). Thus, the overall case cohort will consist of 543 patients, including 66 cases (40 for the standard arm, 26 for the CPX-351 arm). An estimated 33% of the 510 patients included in this analysis will be at high risk; thus, expect 84 high-risk and 171 low-risk patients per arm. Assuming an LVEF standard deviation of 10%, there will be 80% power to determine a difference between treatment arms of 6% LVEF for high-risk patients and 4% LVEF for low-risk patients, accounting for correlations between repeated measures by GEE. Assuming a GLS standard deviation of 2.5%, there will be 80% power to determine a difference between treatment arms of 1.2% GLS for high-risk patients and 1% GLS for low-risk patients.

## 4. Study status

AAML1831 was initially CIRB approved on 4 March 2020 and activated on 20 July 2020. AAML1831 is currently enrolling subjects with newly diagnosed AML at eligible COG sites. Complete study accrual is anticipated near the end of 2025.

## 5. Discussion

AAML1831 is the largest pediatric oncology trial to prospectively assess, in a randomized fashion, the efficacy of two cardioprotective strategies. Detailed cardiac phenotyping of this large cohort with serial echocardiograms and cardiac biomarkers will allow for robust measure of the cardiovascular impacts of conventional daunorubicin with dexrazoxane as compared to liposomal daunorubicin (CPX-351). While prior COG AML drug trials have relied upon limited site-dependent reporting of cardiac adverse events and site-measured echocardiographic measures, AAML1831 is the first phase 3 pediatric oncology trial to employ rigorous core laboratory echocardiographic image quantitation, a methodology that has become more standard in adult oncology trials. Numerous studies have demonstrated the superiority of core lab interpretations for reducing variability and enhancing the precision of echocardiographic measurements (55–59). Thus, the use of central review in the context of AAML1831 will allow for a more precise and accurate assessment of cardiac outcomes across arms to inform the effects of cardioprotective strategies in the modern treatment era. Central review will also enable a detailed assessment of very comprehensive quantitative echocardiographic measures, including cardiac structure, size, and function, throughout AML therapy and in follow-up to advance the understanding of the trajectory of cardiac remodeling and function with AML therapies and identify early predictors of anthracycline cardiomyopathy where early institution of cardioprotective medications may attenuate subsequent LVEF decline and improve the likelihood of recovery (54, 60, 61).

In addition to primary cardioprotection with dexrazoxane and the use of a liposomal encapsulated anthracycline, AAML1831 employs a rigorous, standardized approach to identifying and managing cardiotoxicity that seeks to balance the potentially competing goals of cardiotoxicity reduction and maximizing anthracycline delivery to optimize both leukemia and cardiovascular outcomes (62, 63). Although challenging, the implementation of a standardized echocardiogram acquisition protocol, comprehensive site echocardiography lab training and regular feedback to optimize the quality of images captured, and the prioritization of LVEF quantitated by the biplane Simpson's method will improve the reliability of cardiotoxicity assessment on which sites are basing the safety of ongoing anthracycline administration. In addition, to maximize the delivery of effective cancer therapy, AAML1831 employs a relatively low threshold for anthracycline dose modification (LVEF < 50%), protocol guidance for a repeat echocardiogram in 1 week to assess for recovery prior to withholding anthracyclines, potential reintroduction of anthracycline therapy in patients with transient LVSD, and recommended cardiology consultation with evidence of cardiotoxicity. Although the optimal method to effectively treat leukemia while preserving cardiac function has not been established, careful attention to this balance is warranted, and this study will provide key data to inform evolving cardioprotective strategies.

## 6. Conclusion

Through rigorous study of cardioprotective interventions in a large, randomized phase 3 study, AAML1831 will elucidate the optimal cardioprotective strategy and identify valuable cardiotoxicity risk predictors to improve both cardiovascular and oncologic outcomes in pediatric AML. Given the innovative strategies employed to optimize the reliability and accuracy of cardiac outcome assessments, AAML1831 will serve as a model for cardiotoxicity assessment in future pediatric oncology trials.

## Data Availability

The original contributions presented in the study are included in the article, and further inquiries can be directed to the corresponding author.
